# Locomotor and anxiety symptoms in the late‐onset Alzheimer's disease LOAD2 mouse model with normal age‐related cognitive decline

**DOI:** 10.1002/alz70855_105188

**Published:** 2025-12-24

**Authors:** Shinwon Kang, Ashish Kadia, Junhui Wang, Aram Abbasian, John Georgiou, Graham L Collingridge

**Affiliations:** ^1^ University of Toronto, Toronto, ON, Canada; ^2^ Lunenfeld‐Tanenbaum Research Institute, Toronto, ON, Canada; ^3^ Tanz Centre for Research in Neurodegenerative Diseases, Toronto, ON, Canada

## Abstract

**Background:**

Late‐onset Alzheimer's disease (**LOAD**), the most common form of dementia, arises from complex genetic and environmental interactions. Preclinical models that simulate the slow progression and long prodromal phase of LOAD are critical for identifying early therapeutic targets. The LOAD2 mouse model, developed on the C57BL/6J (B6J) genetic background, integrates key LOAD genetic risk factors: the APOE4 allele, the *Trem2* R47H variant, and humanized *App* mutations within the amyloid‐beta (Aβ) sequence. This study aimed to phenotype LOAD2 mouse behaviours, during ageing.

**Method:**

Behavioural assays were conducted on LOAD2 (JAX strain #030670) and B6J control mice at 18 and 24 months. Both male and female mice were assessed for locomotor activity (open field test), anxiety‐like behaviour (elevated plus maze), spatial working memory (Y‐maze), recognition memory (novel object recognition, novel location discrimination), and contextual memory (fear conditioning).

**Result:**

At 18 months, LOAD2 mice exhibited significantly reduced locomotor activity compared to B6J controls (*p* <0.01). However, at 24 months, this difference diminished as B6J mice displayed an age‐related decline in exploration time. Anxiety‐like behaviour was elevated in 18‐month‐old LOAD2 mice (*p* <0.05), but the difference was no longer evident at 24 months due to increased anxiety levels in aged B6J mice. Spatial working memory, assessed through the Y‐maze task, remained unchanged in LOAD2 mice compared to B6J. Both groups showed impaired novel object and location recognition at 18 and 24 months, suggesting overall age‐related cognitive decline. In contextual fear‐conditioning, LOAD2 mice exhibited higher freezing levels responses compared to B6J controls at 18 months (*p* <0.01). Fear memory was intact since both groups recognized the testing context 24 h later. Subsequently, when the auditory cue was presented in a novel context, both groups showed increased freezing. At 24 months both groups showed comparable performance in contextual and auditory fear memory tests.

**Conclusion:**

LOAD2 mice exhibit early locomotor deficits and heightened anxiety, which are commonly associated with AD. However, both models show similar age‐related declines in exploratory behaviour and recognition memory. Thus, more extensive testing and/or environmental challenges will be needed to detect overt cognitive deficits in LOAD2 mice.

